# Cost-effectiveness of prenatal food and micronutrient interventions on under-five mortality and stunting: Analysis of data from the MINIMat randomized trial, Bangladesh

**DOI:** 10.1371/journal.pone.0191260

**Published:** 2018-02-15

**Authors:** Pernilla Svefors, Katarina Ekholm Selling, Rubina Shaheen, Ashraful Islam Khan, Lars-Åke Persson, Lars Lindholm

**Affiliations:** 1 International Maternal and Child Health, Department of Women’s and Children’s Health, Uppsala University, Uppsala, Sweden; 2 International Centre for Diarrhoeal Disease Research, Bangladesh (icddr,b), Dhaka, Bangladesh; 3 Department of Disease Control, London School of Hygiene and Tropical Medicine, London, United Kingdom; 4 Department of Public Health and Clinical Medicine, Umeå University, Umeå, Sweden; Institut de recherche pour le developpement, FRANCE

## Abstract

**Introduction:**

Nutrition interventions may have favourable as well as unfavourable effects. The Maternal and Infant Nutrition Interventions in Matlab (MINIMat), with early prenatal food and micronutrient supplementation, reduced infant mortality and were reported to be very cost-effective. However, the multiple micronutrients (MMS) supplement was associated with an increased risk of stunted growth in infancy and early childhood. This unfavourable outcome was not included in the previous cost-effectiveness analysis. The aim of this study is to evaluate whether the MINIMat interventions remain cost-effective in view of both favourable (decreased under-five-years mortality) and unfavourable (increased stunting) outcomes.

**Method:**

Pregnant women in rural Bangladesh, where food insecurity still is prevalent, were randomized to early (E) or usual (U) invitation to be given food supplementation and daily doses of 30 mg, or 60 mg iron with 400 μg of folic acid, or MMS with 15 micronutrients including 30 mg iron and 400 μg of folic acid. E reduced stunting at 4.5 years compared with U, MMS increased stunting at 4.5 years compared with Fe60, while the combination EMMS reduced infant mortality compared with UFe60. The outcome measure used was disability adjusted life years (DALYs), a measure of overall disease burden that combines years of life lost due to premature mortality (under five-year mortality) and years lived with disability (stunting). Incremental cost effectiveness ratios were calculated using cost data from already published studies.

**Results:**

By incrementing UFe60 (standard practice) to EMMS, one DALY could be averted at a cost of US$24.

**Conclusion:**

When both favourable and unfavourable outcomes were included in the analysis, early prenatal food and multiple micronutrient interventions remained highly cost effective and seem to be meaningful from a public health perspective.

## Introduction

Impaired antenatal and early life nutrition increases the risk of neonatal deaths and stunting. According to global estimates, maternal and child undernutrition causes 3.1 million child deaths annually [1]and contributes to 35% of disability adjusted life years (DALYs) in children younger than 5 years[2]. The children who survive may suffer long-term consequences such as cognitive impairment[3] and chronic diseases in adulthood[4]. Cost-effective child nutrition interventions are available to improve survival and reduce the risk of stunting[5] (defined as height-for-age less than -2 standard deviations of the WHO growth standards [6]. One example is prenatal nutrition interventions that potentially benefit both the mother and the offspring already at an early stage of development. Prenatal food supplementation, with macronutrients in the form of balanced protein-energy supplements, has resulted in improved foetal growth and reduced perinatal mortality in low- and middle-income countries[7–9]. The optimal timing of food supplementation and the best combination of micronutrients are not, however, fully established. Routinely, supplementation of iron and folate is recommended for pregnant women[10]. Yet, pregnant women in resource-poor settings are frequently deficient in several micronutrients. Under this assumption supplements consisting of multiple micronutrients (MMS) have been introduced.

Reports from studies aimed at studying the efficacy and safety of multiple micronutrient (MMS) supplementation during pregnancy has varied; from encouraging results such as reduced prevalence of low birth weight[11,12] and small-for-gestational-age (SGA) births[13], to suggested increased risks of perinatal and neonatal mortality[14]. No reduction in child mortality or subsequent linear growth failure have been reported in meta-analyses[15]. Most of the published MMS trials have been implemented in resource-poor settings where the population face both macro- and micronutrient deficiencies. In spite of this, MMS has not been given in combination with food supplementation.

In the randomized MINIMat trial (Maternal and Infant Nutrition Interventions, Matlab, isrctn.org identifier ISRCTN16581394), prenatal MMS supplementation was combined with early or usual timing of food supplementation. Previous results have demonstrated substantial reduction in under-five year mortality with MMS combined with early (E) invitation to prenatal food supplementation (i.e. EMMS) compared with the standard 60 mg iron, 400 gm folic acid and invitation to food supplementation at the usual time in pregnancy (UFe60F)[16]. Conversely, infant mortality did not differ across the two timings of food supplementations or the three micronutrient treatment groups. In addition, MMS supplementation was related to more stunting in comparison with Fe60F, while the early invitation to food supplementation compared with the usual timing of the start of supplementation was associated with reduced frequency of stunting before the age of five years[17]. The potential negative effect by prenatal MMS on child linear growth was also supported by lower insulin-like growth factor 1 at 4.5 years in children whose mothers had been allocated to this treatment [18]. This combination of favourable and unfavourable effects illustrates that nutrition interventions may influence multiple outcomes that may be positive as well as negative from a public health perspective. As cost-effectiveness analyses often form part of the basis for decisions on large scale implementation, it is important to include not only primary outcomes but also nutrition intervention’s potential long-term effects.

The MINIMat interventions have been reported to be cost-effective in respect of the primary outcome of reduced infant mortality[19]. However, the potential long-term adverse effects of MMS due to increased stunting were not included in the analysis. The aim of this study is to evaluate the cost-effectiveness of the MINIMat interventions of early food and MMS supplementation compared with usual timing of food and routine iron and folate supplementation, taking into account both premature death (under five-year mortality) and long-term disability (stunting) in terms of cost per disability adjusted life years (DALYs) averted.

## Methods

### Participants, study design and setting

The data originate from the MINIMat trial (Maternal and Infant Nutrition Interventions in Matlab), a factorial randomized trial carried out in Matlab, Bangladesh, a rural sub-district 57 km south-east of the capital Dhaka where food insecurity is still prevalent. At the time when the trial was implemented, the under-five mortality rate in Bangladesh was 74 per 1000 live births [20] and the prevalence of under-five stunting 50% [21]. Pregnant women were recruited to participate in the MINIMat trial from November 2001 to October 2003 and randomized to two daily food supplementation groups, early invitation (E, at about 9 weeks of pregnancy), or usual timing (U, at about 20 weeks of pregnancy) with 600 kcal six days per week. Further, there were three separate micronutrient groups given from 14 weeks of gestation: 60 mg iron and 400 μg folic acid (routine); multiple micronutrients (MMS) with 15 micronutrients, including 30 mg iron and 400 μg folic acid; or 30 mg iron and 400 μg folic acid to control for the lower amount of iron in the MMS supplement. This resulted in six intervention groups, EFe60F (*n* = 738), EMMS (*n* = 740), EFe30F (*n* = 739), UFe60F (*n* = 738), UMMS (*n* = 740) and UFe30F (*n* = 741). In total, 4436 pregnant women participated, giving birth to 3625 live born children from April 2002 to June 2004. Children of mothers participating in the MINIMat trial were followed from birth to 5 years in regards to mortality and to 4.5 years in regards to stunting. The 4.5-year follow-up took place from April 2007 to February 2009.

### Ethics

Written informed consent was obtained from all parents of participating children (separately for the original trial and the two follow-ups). The Ethical Review Committee at the International Centre for Diarrhoeal Disease Research, Bangladesh and the Regional Ethical Review Board at Uppsala University, Sweden approved the study (separately for the original trial and the two follow-ups).

### Cost data

Cost data were retrieved from a recent study by Shaheen et al.[19], which evaluated the cost effectiveness of the MINIMat interventions in regards to reduced infant mortality. Most cost data in the study by Shaheen were available from Khan and Ahmed[22] whereby an “ingredient” or “bottom up” approached was used (enumerators identified and valued each resource required for, and produced by, the nutrition intervention delivery) while data on cost of micronutrients and some staff costs were obtained from the MINIMat project administration. Shaheen et al reported costs for three different delivering modes: 1) NGO; and 2) government-run community nutrition centres (CNCs); as well as a 3) hypothetical highest-cost scenario combining the highest cost for each item presented for NGO-run and government-run CNCs. In this study, we present costs for the highest cost scenario only. The direct costs for the intervention included micronutrient and food supplements, staff, training and administration, capital, community volunteer time, and recurrent activities. Khan and Ahmed [22] annualized all capital costs at a 5% discount rate, further discounting was not done but all costs were adjusted to the price levels for 2015 using the consumer price index [23]. The indirect costs comprised the cost of the participants’ time and estimated as cost of a laborer when labor cost was the lowest. All the cost items except the micronutrient capsules were summarized, representing the cost per pregnant woman for food. The total cost for supplementing one woman was finally calculated by taking the cost per pregnant woman for food multiplied by the adherence to food, plus the cost for micronutrients capsules, also multiplied by the adherence to micronutrients[24].

### Effects

The effect measure is presented as disability adjusted life years (DALYs) averted. DALYs are a measure of overall disease burden that combines years of life lost due to premature mortality (YLLs) and the time spent in an impaired health state, measured as years lived with disability (YLDs). One DALY lost is to be interpreted as one lost year of healthy life. Consequently, one DALY averted is equivalent to the gain of one year of healthy life. Calculating the cost per DALY averted facilitates comparison between different health interventions as well as enables the evaluation of an intervention’s effects on both premature death and long-term disability. The term disability here refers to loss of health due to stunting.

DALYs are typically attributed to a specific disease, health state or age group. As the MINIMat interventions had an effect on both under-five deaths and stunting, DALYs were equal to YLL due to all-cause premature mortality plus YLD due to stunting. DALYs were calculated based on strictly empirical data from the trial for up to five years (DALY5s) and from a lifetime perspective based on secondary data (DALYs). To calculate YLL for DALY5s, we used the exact mortality age of the children who died and subtracted the age at death from 5 years. YLDs were calculated as the sum of months the children had been stunted from birth to 5 years (i.e., they had a height-for-age Z-score below -2 from the WHO reference median) times a disability weight[25]. The children’s length/height were measured 18 times; at birth, every month up to one year, and every three months up to 24 months and then again at 4.5 years. Height-for-age z-scores were calculated by the program WHOAnthro using the WHO growth references[26]. Height-for-age data were linearly interpolated if height measurements were missing in some age intervals between birth and 2 years of age. For example, if the 5-month height measurement was missing for a study participant, the height-for-age z-score was imputed by linear interpolation of the 4- and 6-month height-for age z-scores of that individual. If two or more subsequent values were missing, the values were imputed from the weighted average of the previous and following z-scores. No child had more than 6 subsequent imputed values from birth to 2 years. From 2 to 4.5 years, the height-for-age z-scores were linearly interpolated for every 3-month period to attain a more accurate estimation of months lived with stunting. Children who were stunted at the assessment at 4.5 years of age were assumed to also be stunted at 5 years.

To calculate DALYs from a lifetime perspective, the number of YLL and YLDs were created using the following assumptions. As most child deaths occurred in early infancy, the life expectancy at birth (LE) was used for YLLs. The remaining life expectancy (RLE) at 5 years was used for YLDs. LE and RLE at 5 years in the Matlab area were based on the demographic surveillance data from the year 2003 and 2008, estimated to 69.3 and 69.7 for girls and 67.8 and 66.9 years for boys[27,28]. YLLs and YLDs were discounted with a discount rate of 3%. The average proportion of recovery and incidence of stunting in each intervention group between 4.5 and 10 years were used to calculate YLDs from 5 to 10 years[29]. Stunted children were assumed to be equally affected by stunting throughout infancy, childhood and adulthood. It was assumed that children who were stunted at 4.5 years remained stunted at 5 years and that children who were stunted at 10 years remained so throughout life[30]. Two different disability weights were used. The disability weight attributed to stunting by the global burden of disease study was 0.002[25]. However, to avoid underestimating the potential long-term consequences of cognitive impairment, we chose to include the higher disability weight of 0.024 for development disability due to malnutrition[25]. Finally, the mean of the YLLs and YLDs in each intervention group were summed up to attain the final DALY estimate. The assumptions used to calculate DALYs and DALY5s are summarised in Box 1.

**Box 1. Key assumptions used to calculate DALY5s**

- Children were assumed to grow linearly. Height data were linearly interpolated if height measurements were missing in the age interval between birth and 2 years of age. From 2 to 4.5 years, height-for-age z-scores were imputed every 3 months.
- Duration of disability: Duration of stunting, for up to 5 years, was obtained from observed data. Children who were stunted at 4.5 years were assumed to be stunted at 5 years.
- Disability weights used: 0.002 (stunting) and 0.024 (developmental delay). Stunted children were assumed to be equally affected by the disability caused by stunting throughout infancy and childhood.
- Discount rate: No discounting.

**Key assumptions used to calculate DALYs**

- Duration of disability: Duration of stunting, for up to 5 years, was obtained from observed data. From 5 to 10 years, the average proportion of recovery and incidence of stunting in each intervention group between 4.5 and 10 were used. Children who were stunted at 4.5 years were assumed to be stunted at 5 years, and children who were stunted at 10 years were assumed to continue being stunted throughout life.
- Disability weights used: 0.002 (stunting) and 0.024 (developmental delay). Stunted children were assumed to be equally affected by the disability caused by stunting throughout infancy, childhood and adulthood.
- Discount rate: 3%.

Life expectancy: LE at birth for YLL, and RLE at 5 years for YLD. Discounted LE and RLE for girls; 28.66 and 29.03 years. Discounted LE and RLE for boys; 28.16 and 28.84

### Analyses

To obtain incremental cost effectiveness ratios (ICERs) for DALYs averted, we calculated the costs for supplementing 1 pregnant woman following the regimes in each intervention arm. These were then sorted according to ascending costs and dominating alternatives were excluded (i.e., alternatives with both a higher cost and a higher DALY estimate). This procedure resulted in three remaining intervention arms. Lastly, we calculated the increment in costs and DALYs across these three arms and divided the cost differences by the differences between DALYs, which gave the ICERs.

## Results

**Fig 1:**
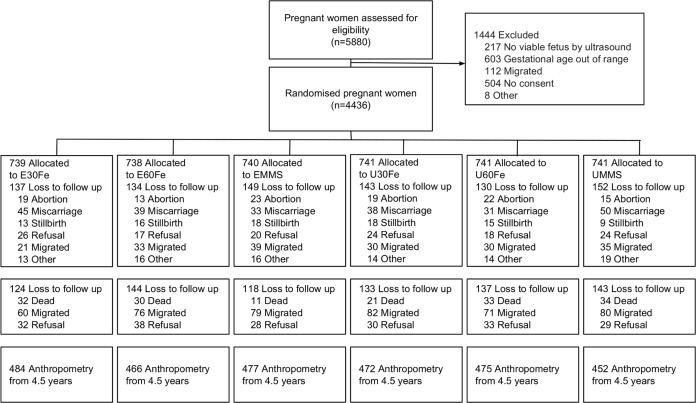
Flow diagram of women and infants participating in the MINIMat trial. E = Early invitation food supplementation, U = Usual invitation food supplementation; 30F = 30 mg iron and 400 μg of folic acid; 60F = 60 mg iron and 400 μg of folic acid; MMS = multiple micronutrients, 15 micronutrients including 30 mg iron and 400 μg of folic acid.

There were 4,436 women enrolled into the MINIMat trial, of whom 845 were lost to follow-up before delivery, mainly due to fetal loss, outmigration or because they withdrew their consent. Of the 3,625 live born children 2,851 had anthropometry at 4.5 years (Fig 1). Background characteristics of the mothers and children of the MINIMat trial is presented in Table 1. The mothers had an average of 5.2 years (SD 4.1) of education and 40% of the mothers belonged to the lowest socioeconomic category. The mean maternal height was 149.8 cm (SD 5.3), mean maternal weight was 45.3 kg (SD 6.8), and one third of the of the mothers were underweight, with a body mass index of less than 18.5 at enrolment. The average birth length and weight of the children were 47.7 cm (SD 2.2) and 2,694 grams (SD 410) respectively. More than half of the children (59%) were born small for gestational age (SGA). The baseline characteristics were similar across the six intervention groups.

**Table 1:** Baseline characteristics of mothers at 8 weeks of gestation and children at birth participating in the MINIMat trial, Bangladesh.

CHARACTERISTICS		n	%
Mothers			
Age			
	<20	916/3267	16.0
	20–29	1862/3267	57.0
	≥30	882/3267	27.0
BMI at 8 weeks			
	<18.5	916/3255	28.1
	≥18.5	2339/3255	71.9
Education			
	No Education	1016/3267	31.1
	Can read and write	2251/3267	68.9
SES category			
	Low	1329/3267	40.7
	Middle	651/3267	19.9
	High	1287/3267	39.4
Children			
Sex			
	Girl	1605/3267	49.1
SGA ^**1**^		1928/3267	59.0
LBW^**2**^		997/3267	30.5
Preterm ^**3**^		258/3267	7.9

^**1**^Small for Gestational Age (Birth weight below reference for gestational week at birth (30)

^**2**^Low Birth Weight (<2500 g)

^**3**^Born before 37 weeks of gestation

### Costs

Adherence levels and costs for a hypothetical highest-cost delivery scenario are presented in Table 2. Using these figures, supplementing 1 pregnant woman would cost US$82.12, US$79.46, US$86.19, US$55.34, US$54.42, and US$56.73 for E30F, E60f, EMMS, U30F, U60F and UMMS, respectively.

**Table 2:** Adherence to and cost of food supplements and micronutrient capsules and cost for supplementing one woman.

	Adherence to food packets	Adherence to micronutrient capsules	Cost for supplementing one woman^1^^,^^2^^,^^3^
**EFe30**	91	109	82.12
**EFe60**	88	113	79.47
**EMMS**	94	107	86.19
**UFe30**	61	117	55.34
**UFe60**	60	113	54.42
**UMMS**	61	110	56.73

Adherence to and cost of food supplements and micronutrient capsules and for supplementing one woman in the MINIMat trial, Bangladesh.

**1** US dollars adjusted to price levels 2015 using consumer price index (World bank)

**2** Cost per pregnant woman for food 0.895, cost for micronutrient capsule MMS 0.020, iron-folic acid 30/60 0.0065

**3** Cost for supplementing one woman (cost for food packets * adherence to food packets) + (cost for micronutrient capsule * adherence to micronutrient capsules)

### YLLs, YLSs, YLDs and DALYs

Years lost to premature mortality (YLL), years lived with stunting (YLS), years lived with disability (YLD) and disability adjusted life years (DALYs) lost, for up to 5 years and calculated from a lifetime perspective, are presented in Tables 3 and 4. The average number of years lost to premature mortality was lowest in the EMMS group and highest in the UMMS group, both for up to five years and from a lifetime perspective (0.080, 0.27 YLLs up to 5 years and 0.62, 2.09 YLLs for the lifetime). The UMMS group also had the highest estimated number of years lived with stunting in both scenarios (1.68, 12.92 YLS). The least number of years were lived with stunting in the E60Fe group, for up to 5 years, and in the U30Fe group over a lifetime. The DALY5s and DALYs lost were lowest in the EMMS group (0.08 and 0.64) and highest in the UMMS group (0.28 and 2.12), irrespective of disability weight used.

**Table 3:** YLL, YLS, YLD and DALY estimates for up to 5 years within the MINIMat study.

	YLL ^1^	YLS^2^	YLD^3^		DALY5^4^	
	Mean (sd)	Mean (sd)	Mean (sd) 0.002	Mean (sd) 0.024	Mean (sd) 0.002	Mean (sd) 0.024
**E30Fe**	0.2272(1.00)	1.54 (1.82)	0.0031 (0.0036)	0.0369(0.04)	0.2303(1.00)	0.2641(0.99)
**E60Fe**	0.2216(1.00)	1.34(1.73)	0.0027 (0.0035)	0.0322(0.04)	0.2243(1.00)	0.2539(0.99)
**EMMS**	0.0800(0.61)	1.56(1.83)	0.0031 (0.0037)	0.0375(0.04)	0.0831(0.61)	0.1174(0.61)
**U30Fe**	0.1690(0.87)	1.59(1.86)	0.0032 (0.0037)	0.0383(0.05)	0.1720(0.87)	0.2069(0.86)
**U60Fe**	0.2510(1.05)	1.59(1.90)	0.0033 (0.0038)	0.0383(0.05)	0.2542(1.05)	0.2893(1.04)
**UMMS**	0.2737(1.10)	1.68(1.93)	0.0034 (0.0039)	0.0404(0.05)	0.2770(1.10)	0.3140(1.09)

**1** Years of life lost due to premature mortality for up to 5 years

**2** Years lived with stunting, up to 5 years

**3** Years lived with disability, years lived with stunting multiplied by the disability weight of 0.002 and 0.024

**4** YLL+YLD for up to five years

**Table 4:** YLL, YLS, YLD and DALY estimates within the MINIMat study.

	YLL^1^	YLS^2^	YLD^3^		DALY^4^	
	Mean (sd)	Mean	Mean 0.002	Mean 0.024	Mean 0.002	Mean 0.024
**E30Fe**	1.7162(7.37)	11.7417	0.0235	0.2818	1.7397	1.9980
**E60Fe**	1.7054(7.34)	10.1577	0.0203	0.2438	1.7257	1.9492
**EMMS**	0.6177(4.49)	11.4962	0.0230	0.2759	0.6406	0.8936
**U30Fe**	1.2825(6.42)	9.7237	0.0194	0.2334	1.3019	1.5159
**U60Fe**	1.9173(7.77)	11.8206	0.0236	0.2837	1.9409	2.2010
**UMMS**	2.0938(8.09)	12.9199	0.02584	0.3101	2.1196	2.4039

**1** Years of life lost due to premature mortality when LE at birth discounted at 3%, present value 29.04, 28.84 for women and men.

**2** Years lived with stunting when RLE at five years discounted at 3%, present value 28.66, 28.16 for women and men.

**3** Years lived with disability, years lived with stunting multiplied by the disability weight of 0.002 and 0.024, respectively

**4** YLL+YLD

### Incremental cost-effectiveness ratios

Incremental costs, incremental DALYs and incremental cost-effectiveness ratios (ICER) are presented in Table 5 for up to 5 years, and in Table 6 from a lifetime perspective. After excluding the alternatives that had both a higher cost and a higher DALY estimate than the standard alternative of U60Fe, (i.e., the dominated alternatives), we ended up with three remaining intervention arms, EMMS, U30Fe and U60Fe, for both scenarios. A total of 0.1 DALY5s and 0.7 DALYs per child were averted by switching to U30Fe, and 0.2 DALY5s and 1.3 DALYs per child were averted by switching to EMMS. The ICER for one extra DALY5 averted by switching from UFe60 to UFe30 was US$11.5, and from UFe60 to EMMS, US$186.9. The corresponding figures for one DALY averted were US$1.4 and US$24.4, respectively. When using the higher disability weight of 0.024, the ICER increased by US$2 to US$26.3 per DALY averted (S1 Table).

**Table 5:** Incremental cost-effectiveness ratios for cost per DALY5s averted.

	Cost/woman	DALYs/child^1^	Comparison	Incremental cost	Incremental DALY5s	ICER^2^
**U60fe**	54.4157	0.2542				
**U30Fe**	55.3364	0.1720				
**UMMS**	56.7306	0.2770				
**E60Fe**	79.4645	0.2243				
**E30Fe**	82.1221	0.2303				
**EMMS**	86.1935	0.0831				
**Incremental cost-effectiveness ratios after excluding dominated alternatives**						
**U60fe**	54.4157	0.2542	U60fe-U30Fe	0.92	0.08	11.5
**U30Fe**	55.3364	0.1720	U30Fe-EMMS	30.86	0.09	342.9
**EMMS**	86.1935	0.0831	60Fe-EMMS	31.78	0.17	186.9

Incremental cost-effectiveness ratios for cost per DALY averted for the different MINIMat prenatal food and micronutrient supplementation arms.

^1^ Disability weight 0.002

^2^ Incremental cost/Incremental DALYs

**Table 6:** Incremental cost-effectiveness ratios for cost per DALY averted.

	Cost/woman	DALYs /child^1^	Comparison	Incremental cost	Incremental DALYs	ICER^2^
**U60fe**	54.4157	1.9409				
**U30Fe**	55.3364	1.3019				
**UMMS**	56.7306	2.1196				
**E60Fe**	79.4645	1.7257				
**E30Fe**	82.1221	1.7397				
**EMMS**	86.1935	0.6406				
**Incremental cost-effectiveness ratios after excluding dominated alternatives**						
**U60fe**	54.4157	1.9409	60fe-U30Fe	0.92	0.64	1.4
**U30Fe**	55.3364	1.3019	30Fe-EMMS	30.86	0.66	46.8
**EMMS**	86.1935	0.6406	60Fe-EMMS	31.78	1.30	24.4

Incremental cost-effectiveness ratios for cost per DALY averted for the different MINIMat prenatal food and micronutrient supplementation arms.

^1^ Disability weight 0.002

^2^ Incremental cost/Incremental DALYs

## Discussion

### Main findings and consequences for public health policy

When favourable (decreased under-five year mortality) and unfavourable (increased stunting), outcomes of the MINIMat trial were included in the analysis the implementation of EMMS supplementation, when compared to U60Fe, remained highly cost-effective. The incremental cost effectiveness ratio per 5 years and lifetime DALY averted of US$187 and US$24 both fall well below Bangladesh’s per-capita gross national income (US$1190 in 2015) and compare favourably to corresponding interventions evaluated elsewhere in South Asia[31–33]. In a community-based newborn care intervention in Bangladesh, one DALY could be averted at a cost of US$103[34], and, in Nepal, a participatory women’s group intervention reported an ICER of US$211[32] per life year saved, which is much higher than our estimates.

The main aim of this study was to evaluate the cost of switching from the routine prenatal supplementation of 60 mg iron and folate plus the usual timing (week 20) of daily food supplements (U60Fe) to the potentially superior multiple micronutrients plus an early invitation (week 9) to food supplementation (EMMS) including both favourable and unfavourable outcomes, but all the intervention arms were included in the analysis. It turned out that switching from U60Fe to U30Fe had the lowest ICER, at US$1.4 per DALY averted. As the cost for the two interventions were similar, the ICER for switching from U60Fe to U30Fe was mainly driven by the slightly lower DALYs in the U30Fe group due to primarily lower YLL but also lower YLD estimates. The fact that U30Fe would have a lower YLL estimate was somewhat expected as the U30Fe group had a lower mortality hazard ratio in the survival analysis by Persson et al. [16] than U60Fe (0.67), although not statistically significant (CI 0.69–1.7)[16]. The WHO recommendations for iron supplementation during pregnancy is 30–60 mg per day, the higher dose recommended in settings where anaemia in pregnant women is widespread, such as rural Bangladesh, where the prevalence of anaemia in pregnant women is estimated to be 49.6%[35]. However, the prevalence of iron deficiency in Bangladesh and in the MINIMat population is reportedly low due to high levels of iron in the groundwater[36,37], suggesting that other micronutrient deficiencies or infections might be important causes. Further, superfluous supplementation of iron could contribute to an excess iron load with potential health hazards[38,39].

Although switching from U60fe to U30Fe had the lowest ICER, EMMS had the lowest DALY estimate. Switching from U60Fe to EMMS would avert twice as many DALYs as switching to U30Fe. As the ICER of US$24 can be considered highly cost-effective and affordable in a low-resource setting, switching from U60Fe to EMMS is viable both from a public health and economic perspective. In addition, as mentioned above, when modelled, the estimates that generated the lower YLLs and YLDs in the U30Fe intervention group were not statistical significant[16].

The lower DALYs in the EMMS group was driven primarily by the lower YLLs as the difference in YLD between U60Fe and EMMS was minor. Meta-analyses of prenatal multiple micronutrient supplementation trials have reported limited increases in birth weight in comparison with iron-folic acid supplementation alone[11,12], and some reduction in the prevalence of small-for-gestational-age births[13], but no reduction in the risk of child mortality or subsequent linear growth failure[15]. A recent Cochrane review of prenatal MMS supplementation found high-quality evidence for a small reduction of the occurrence of stillbirth outcomes but no effect on perinatal or neonatal mortality[40]. The newly updated WHO guidelines on antenatal care do not recommend multiple micronutrients for pregnant women[41].

What makes the MINIMat interventions different to the trials included in the above-mentioned studies is that the multiple micronutrient supplementation was combined with food supplementation early in pregnancy and the reduction in mortality was only seen as a combined effect of early food supplementation and MMS. MMS given without food supplementation in *early* pregnancy (UMMS) had the highest DALY estimates as a result of both the highest number of YLLs and YLDs. Further, in a recently published study the MINIMat trial children allocated to the multiple micronutrient supplementation displayed an unfavourable metabolic profile at 4,5 years with lower levels of IGF and HDL[18]. These results indicate positive outcomes of providing MMS only when it is combined with increased macronutrient intake that is initiated in early pregnancy.

### Generalisability

The MINIMat trial was a community-based trial, conducted in an area with a well-established health and demographic surveillance system and an excellent research infrastructure that fulfils the prerequisites for obtaining high-quality data. The randomised design minimised the risk of potential confounding and double masking of the micronutrient intervention reduced the risk of reporting or observation bias.

In the area where the trial was conducted, women are still frequently exposed to food insecurity and enter pregnancy deficient in several micronutrients. This makes the ICERs estimates valid to other rural areas in Bangladesh and to similar settings in other countries that struggle with problems of widespread maternal malnutrition. The cost data retrieved from the study by Shaheen et al. were collected within the same time-frame and in a situation similar to that of the MINIMat trial, which is why we believe they reasonably represent costs associated with the MINIMat interventions. Shaheen et al. presented three different delivery modes, resulting in three cost scenarios, NGO-run clinics, governmental-run clinics and a highest-cost delivery mode where the highest costs for different items from NGO- and government-run clinics were combined. As we used the highest cost scenario in our analysis, our generated ICERs might be somewhat overestimated.

### Methodological considerations

Most nutrition interventions have multiple objectives and outcomes. In this study, we included two outcomes; premature mortality, and disability caused by stunting, aware that this do not represent all potential benefits or harms of the intervention[42].

When evaluating the cost-effectiveness of multiple outcomes, the issue of assigning values to the different outcomes becomes essential. The disability weight attributed to stunting by the global burden of disease study, 0.002, is small. In order to avoid underestimating the long-term effects of stunting, we chose to include the larger disability weight attributed to development delay due to malnutrition, although not all stunted children will suffer from cognitive impairment. When using the higher disability weight, the ICER only increased from US$24 to US$26 as differences in YLS between the intervention groups were small. The differences in YLS and YLL between the intervention group with the lowest and highest estimate were 3.2 YLS (UMMS-U30Fe) and 1.47 YLL (UMMS-EMMS) respectively. Thus, for the differences in YLS to change the ranking of the highest and lowest DALY estimate, the disability weight would have to be at least as high as 0.4 (1.47/3.2), equal to multiple sclerosis or bipolar disorder[25]. Further, the disability weights, and thus the cost-effectiveness analysis, does not consider stunting’s additional health and economic consequences for the individual and community, such as impaired pregnancy outcomes for women[43], higher risk of non-communicable disease[44], fewer years of schooling [45]and lower earnings[3]. However, the conclusion that switching from Ufe60 to EMMS is highly cost-effective would not change if you increase the impact of stunting, as U60Fe had higher YLS estimates than EMMS. The economic impacts of stunting might better be valued in monetary units and by using cost-benefit analysis.

Another key assumption associated with the impact of stunting was that all children who were stunted at 10 years continued to be stunted throughout life, based on the fact that adult stature is strongly associated with pre-pubertal height [30]. This assumption applies equally for all intervention groups, and hence, does not significantly affect the difference in DALYs or the ICERs.

The estimate of US$187 per DALY averted for up to 5 years was based on strictly observed data from the trial and was not subject to any assumptions except the disability weight for stunting. However, by not considering the number of life years lost to premature mortality and disability beyond the age of 5 years, the intervention is at a disadvantage when compared with alternatives, which are usually analysed from a lifetime perspective.

## Conclusions

Including stunting as an outcome did not alter the conclusion that the MINIMat interventions of early prenatal food and MMS supplementation were highly cost-effective in a population where maternal undernutrition is still common. The ICER of US$24 for switching from invitation to food supplementation at the usual time in pregnancy and iron-folic acid supplementation to an early initiation of food supplementation combined with MMS can be considered affordable in a low-resource setting and viable from both a public health and economic perspective. These results can hopefully inspire others to consider multiple outcomes of nutritional interventions in economic evaluations and help to support decision-makers in the prioritizing of financing for nutrition interventions targeting pregnant women’s health.

## Supporting information

S1 Table Incremental cost-effectiveness ratios (ICERs) for cost per DALY averted for the different MINIMat prenatal food and micronutrient supplementation arms, disability weight 0.024. (DOCX)

S1 Dataset Anonymized data set. (SAV)

## References

[1] Black RE, Victora CG, Walker SP, Bhutta ZA, Christian P, de Onis M, et al Maternal and child undernutrition and overweight in low-income and middle-income countries. Lancet. 2013;382:427–51. doi: 10.1016/S0140-6736(13)60937-X 23746772 10.1016/S0140-6736(13)60937-X

[2] Black RE, Allen LH, Bhutta ZA, Caulfield LE, de Onis M, Ezzati M, et al Maternal and child undernutrition: global and regional exposures and health consequences. Lancet. 2008;371:243–60. doi: 10.1016/S0140-6736(07)61690-0 18207566 10.1016/S0140-6736(07)61690-0

[3] Grantham-McGregor S, Cheung YB, Cueto S, Glewwe P, Richter L, Strupp B. Developmental potential in the first 5 years for children in developing countries. Lancet. 2007;369:60–70. doi: 10.1016/S0140-6736(07)60032-4 17208643 10.1016/S0140-6736(07)60032-4 PMC2270351

[4] Prentice AM, Moore SE. Early programming of adult diseases in resource poor countries. Arch Dis Child.2005;90:429–32. doi: 10.1136/adc.2004.059030 15781942 10.1136/adc.2004.059030 PMC1720333

[5] Bhutta ZA, Ahmed T, Black RE, Cousens S, Dewey K, Giugliani E, et al What works? Interventions for maternal and child undernutrition and survival. Lancet. 2008;371:417–40. doi: 10.1016/S0140-6736(07)61693-6 18206226 10.1016/S0140-6736(07)61693-6

[6] de Onis M, World Health Organization. WHO child growth standards: length/height-for-age, weight-for-age, weight-for-length, weight-for-height and body mass index-for-age: methods and development Geneva: World Health Organization; 2006.

[7] Kramer MS, Kakuma R. Energy and protein intake in pregnancy. Cochrane Database Syst Rev. 2003;4:CD000032 doi: 10.1002/14651858.CD000032 10.1002/14651858.CD000032 14583907

[8] Ceesay SM, Prentice AM, Cole TJ, Foord F, Poskitt EME, Weaver LT, Whitehead RG. Effects on birth weight and perinatal mortality of maternal dietary supplements in rural Gambia: 5 year randomised controlled trial. BMJ. 1997;315:786–90. 9345173 10.1136/bmj.315.7111.786 PMC2127544

[9] Imdad A, Bhutta ZA. Effect of balanced protein energy supplementation during pregnancy on birth outcomes. BMC Public Health.2011;11:S17 doi: 10.1186/1471-2458-11-S3-S17 21501434 10.1186/1471-2458-11-S3-S17 PMC3231890

[10] World Health Organization. WHO Guideline: Daily iron and folic acid supplementation in pregnant women Geneva: World Health Organization; 2012. 23586119

[11] Fall CHD, Fisher DJ, Osmond C, Margetts BM. Multiple Micronutrient Supplementation during Pregnancy in Low-Income Countries: A Meta-Analysis of Effects on Birth Size and Length of Gestation. Food Nutr Bull. 2009;30:S533–46. doi: 10.1177/15648265090304S408 20120795 10.1177/15648265090304S408 PMC3541502

[12] Margetts BM, Fall CHD, Ronsmans C, Allen LH, Fisher DJ, Maternal Micronutrient Supplementation Study Group. Multiple Micronutrient Supplementation during Pregnancy in Low-Income Countries: Review of Methods and Characteristics of Studies Included in the Meta-Analyses. Food Nutr Bull. 2009;30:S517–26. doi: 10.1177/15648265090304S406 20120793 10.1177/15648265090304S406

[13] Haider BA, Yakoob MY, Bhutta ZA. Effect of multiple micronutrient supplementation during pregnancy on maternal and birth outcomes. BMC Public Health. 2011;11:S19 doi: 10.1186/1471-2458-11-S3-S19 21501436 10.1186/1471-2458-11-S3-S19 PMC3231892

[14] Christian P, Osrin D, Manandhar DS, Khatry SK, de L Costello AM, West KP. Antenatal micronutrient supplements in Nepal. Lancet. 2005;366:711–2. doi: 10.1016/S0140-6736(05)67166-8 16125578 10.1016/S0140-6736(05)67166-8

[15] Devakumar D, Fall CHD, Sachdev HS, Margetts BM, Osmond C, Wells JCK, et al Maternal antenatal multiple micronutrient supplementation for long-term health benefits in children: a systematic review and meta-analysis. BMC Medicine. 2016;14:90 doi: 10.1186/s12916-016-0633-3 27306908 10.1186/s12916-016-0633-3 PMC4910255

[16] Persson LÅ, Arifeen S, Ekström E-C, Rasmussen KM, Frongillo EA, Yunus M, et al Effects of Prenatal Micronutrient and Early Food Supplementation on Maternal Hemoglobin, Birth Weight, and Infant Mortality Among Children in Bangladesh: The MINIMat Randomized Trial. JAMA.2012;307:2050–9. doi: 10.1001/jama.2012.4061 22665104 10.1001/jama.2012.4061

[17] Khan AI, Kabir I, Hawkesworth S, Ekström E-C, Arifeen S, Frongillo EA, et al Early invitation to food and/or multiple micronutrient supplementation in pregnancy does not affect body composition in offspring at 54 months: follow-up of the MINIMat randomised trial, Bangladesh. Matern Child Nutr. 2012;11:385–97. doi: 10.1111/mcn.12021 23241449 10.1111/mcn.12021 PMC6860263

[18] Ekström E-C, Lindström E, Raqib R, Arifeen El S, Basu S, Brismar K, et al Effects of prenatal micronutrient and early food supplementation on metabolic status of the offspring at 4.5 years of age. The MINIMat randomized trial in rural Bangladesh. Int J Epidemiol. 2016;45: 1656–1667. doi: 10.1093/ije/dyw199 27694568 10.1093/ije/dyw199 PMC5100620

[19] Shaheen R, Persson LÅ, Ahmed S, Streatfield PK, Lindholm L. Cost-effectiveness of invitation to food supplementation early in pregnancy combined with multiple micronutrients on infant survival: analysis of data from MINIMat randomized trial, Bangladesh. Ann Hum Biol. 2015;15:1–8. 10.1186/s12884-015-0551-y PMC4445523 26018633

[20] Mortality rate, under-5 (per 1,000 live births) | Data. In: data.worldbank.org [Internet]. [cited 30 Nov 2017]. Available: https://data.worldbank.org/indicator/SH.DYN.MORT

[21] 2014 Nutrition Country Profile: Bangladesh. In: globalnutritionreport.org [Internet]. [cited 30 Nov 2017]. Available: http://www.globalnutritionreport.org/files/2014/11/gnr14_cp_bangladesh.pdf

[22] Khan MM, Ahmed S. Relative efficiency of government and non-government organisations in implementing a nutrition intervention programme–a case study from Bangladesh. Public Health Nutr. Cambridge University Press; 2003;6: 19–24. doi: 10.1079/PHN2002359 12581461 10.1079/PHN2002359

[23] The World Bank. Consumer Price Index 1986–2015, Bangladesh. 2015. Available from: http://data.worldbank.org/indicator/FP.CPI.TOTL?locations=BD Accessed 26 Feb 2017.

[24] Shaheen R, Streatfield PK, Naved RT, Lindholm L, Persson LÅ. Equity in adherence to and effect of prenatal food and micronutrient supplementation on child mortality: results from the MINIMat randomized trial, Bangladesh. BMC Public Health. 2014;14:5 doi: 10.1186/1471-2458-14-5 24393610 10.1186/1471-2458-14-5 PMC3893435

[25] World Health Organization. Global Burden of Disease 2004 update: Disability Weights for Diseases and Conditions. Geneva: World Health Organization; 2004.

[26] de Onis M, World Health Organization. WHO IRIS: WHO child growth standards: length/height-for-age, weight-for-age, weight-for-length, weight -for-height and body mass index-for-age: methods and development. 2006.

[27] International Centre for Diarrhoeal Disease Research, Bangladesh (ICDDR B). Health and Demographic Surveillance System–Matlab Registration of health and demographic events 2003. Dhaka: Centre for Health and Population Research; 2005.

[28] International Centre for Diarrhoeal Disease Research, Bangladesh (ICDDR B). Health and Demographic Surveillance System–Matlab Registration of health and demographic events 2008. Dhaka: Centre for Health and Population Research; 2010.

[29] Svefors P, Rahman A, Ekström E-C, Khan AI, Lindström E, Persson LÅ, et al Stunted at 10 Years. Linear Growth Trajectories and Stunting from Birth to Pre-Adolescence in a Rural Bangladeshi Cohort. PLoS ONE. 2016;11:e0149700–18. doi: 10.1371/journal.pone.0149700 26934484 10.1371/journal.pone.0149700 PMC4775024

[30] Stein AD, Wang M, Martorell R, Norris SA, Adair LS, Bas I, et al Growth patterns in early childhood and final attained stature: Data from five birth cohorts from low- and middle-income countries. Am J Hum Biol. 2009;22:353–9. 10.1002/ajhb.20998 PMC3494846 19856426

[31] Puett C, Sadler K, Alderman H, Coates J, Fiedler JL, Myatt M. Cost-effectiveness of the community-based management of severe acute malnutrition by community health workers in southern Bangladesh. Health Policy Plan. 2013;28:386–99. doi: 10.1093/heapol/czs070 22879522 10.1093/heapol/czs070

[32] Borghi J, Thapa B, Osrin D, Jan S, Morrison J, Tamang S, et al Economic assessment of a women’s group intervention to improve birth outcomes in rural Nepal. Lancet. 2005;366:1882–4. doi: 10.1016/S0140-6736(05)67758-6 16310555 10.1016/S0140-6736(05)67758-6

[33] Menon P, McDonald CM, Chakrabarti S. Estimating the cost of delivering direct nutrition interventions at scale: national and subnational level insights from India. Matern Child Nutr. 2016;12:169–85. doi: 10.1111/mcn.12257 27187914 10.1111/mcn.12257 PMC6680110

[34] LeFevre AE, Shillcutt SD, Waters HR, Haider S, Arifeen El S, Mannan I, et al Economic evaluation of neonatal care packages in a cluster-randomized controlled trial in Sylhet, Bangladesh. Bull World Health Organ. 2013;91:736–45. doi: 10.2471/BLT.12.117127 24115797 10.2471/BLT.12.117127 PMC3791651

[35] National Institute of Population Research and Training (NIPORT), Mitra and Associates, ICF International. Bangladesh Demographic and Health Survey 2011. NIPORT, Mitra and Associates, and ICF International: Dhaka, Bangladesh, and Calverton USA; 2011.

[36] Ziaei S, Rahman A, Raqib R, Lönnerdal B, Ekström EC. A Prenatal Multiple Micronutrient Supplement Produces Higher Maternal Vitamin B-12 Concentrations and Similar Folate, Ferritin, and Zinc Concentrations as the Standard 60-mg Iron Plus 400-g Folic Acid Supplement in Rural Bangladeshi Women. J Nutr. 2016; 146:2520–2329. doi: 10.3945/jn.116.235994 27798335 10.3945/jn.116.235994 PMC5118763

[37] Rahman S, Ahmed T, Rahman AS, Alam N, Ahmed AS, Ireen S, et al Determinants of iron status and Hb in the Bangladesh population: the role of groundwater iron. Public Health Nutr. 2016;19:1862–74. doi: 10.1017/S1368980015003651 26818180 10.1017/S1368980015003651 PMC10270950

[38] Tiwari AKM, Mahdi AA, Chandyan S, Zahra F, Godbole MM, Jaiswar SP, et al Oral iron supplementation leads to oxidative imbalance in anemic women: A prospective study. Clin Nutr ESPEN. 2011;30:188–93. 10.1016/j.clnu.2010.08.001 20888091

[39] Sazawal S, Black RE, Ramsan M, Chwaya HM, Stoltzfus RJ, Dutta A, et al Effects of routine prophylactic supplementation with iron and folic acid on admission to hospital and mortality in preschool children in a high malaria transmission setting: community-based, randomised, placebo-controlled trial. Lancet. 2006;367:133–43. doi: 10.1016/S0140-6736(06)67962-2 16413877 10.1016/S0140-6736(06)67962-2

[40] Haider BA, Bhutta ZA. Multiple-micronutrient supplementation for women during pregnancy. Cochrane Database Syst Rev. 2015;11:CD004905. 10.1002/14651858.CD004905.pub4 PMC6464025 26522344

[41] World Health Organization. WHO recommendations on antenatal care for a positive pregnancy experience Geneva: World Health Organization; 2016. 28079998

[42] Persson LÅ, Arifeen El S, Khan A, Rahman A, Ekström E-C. Effects of early prenatal food supplementation and multiple micronutrients on under-five survival, linear growth, metabolic markers and blood pressure up to 10 years of age. The MINIMat trial in rural Bangladesh. The FASEB Journal. 2017;31: 786.35–786.35.

[43] Özaltin E. Association of Maternal Stature With Offspring Mortality, Underweight, and Stunting in Low- to Middle-Income Countries. JAMA. 2010;303:1507–21. doi: 10.1001/jama.2010.450 20407060 10.1001/jama.2010.450 PMC3100588

[44] Stein AD, Thompson AM, Waters A. Childhood growth and chronic disease: evidence from countries undergoing the nutrition transition. Matern Child Nutr. 2005;1:177–84. doi: 10.1111/j.1740-8709.2005.00021.x 16881898 10.1111/j.1740-8709.2005.00021.x PMC6860951

[45] Martorell R, Horta BL, Adair LS, Stein AD, Richter L, Fall CHD, et al Weight gain in the first two years of life is an important predictor of schooling outcomes in pooled analyses from five birth cohorts from low- and middle-income countries. J. Nutr. 2010;140:348–54. doi: 10.3945/jn.109.112300 20007336 10.3945/jn.109.112300 PMC2806888

