# Supplementary material for: Cost-effectiveness of prenatal food and micronutrient interventions on under-five mortality and stunting: Analysis of data from the MINIMat randomized trial, Bangladesh
Source: PLoS One. 2018 Feb 15;13(2):e0191260. doi: 10.1371/journal.pone.0191260 (PMC5814099; doi:10.1371/journal.pone.0191260)
Supplement: S1 Table — (DOCX) [file pone.0191260.s001.docx]

| **S1 Table.** Incremental cost-effectiveness ratios (ICERs) for cost per DALY averted for the different MINIMat prenatal food and micronutrient supplementation arms, disability weight 0.024. | | | | | | |
| --- | --- | --- | --- | --- | --- | --- |
|  | **Cost/woman** | **DALYs /child** | **Comparison** | **Incremental cost** | **Incremental DALYs** | **ICER** |
| **U60fe** | 54.4157 | 2.2010 | 1.9409 |  |  |  |
| **U30Fe** | 55.3364 | 1.5159 | 1.3019 |  |  |  |
| **UMMS** | 56.7306 | 2.4039 | 2.1196 |  |  |  |
| **E60Fe** | 79.4645 | 1.9492 | 1.7257 |  |  |  |
| **E30Fe** | 82.1221 | 1.9980 | 1.7397 |  |  |  |
| **EMMS** | 86.1935 | 0.8936 | 0.6406 |  |  |  |
| **Incremental cost-effectiveness ratios after excluding dominated alternatives** | | | | | | |
| **U60fe** | 54.4157 | 2.2010 | 60fe-U30Fe | 0.9207 | 0.6851 | 1.3439 |
| **U30Fe** | 55.3364 | 1.5159 | 30Fe-EMMS | 30.8571 | 0.6223 | 49.556 |
| **EMMS** | 86.1935 | 0.8936 | 60Fe-EMMS | 31.7778 | 1.2074 | 26.319 |
| Incremental cost-effectiveness ratios for cost per DALY averted for the different MINIMat prenatal food and micronutrient supplementation arms.  ^1^ Disability weight 0.024  ^2^ Incremental cost/Incremental DALYs | | | | | | |
